# Supplementary material for: Force perceptual bias caused by muscle activity in unimanual steering
Source: PLoS One. 2019 Oct 22;14(10):e0223930. doi: 10.1371/journal.pone.0223930 (PMC6804982; doi:10.1371/journal.pone.0223930)
Supplement: Attachment — Submitted filename: Response to Reviewers.docx [file pone.0223930.s001.docx]

**ID:	PONE-D-19-16009**

**Title:	Force perceptual bias caused by muscle activity in unimanual steering**

The authors would like to thank the editor and the reviewers for their valuable and constructive comments. The paper has been revised by considering the suggestions, in particular, the following issues. Each part revised in the resubmitted article was highlighted with the answer number, which corresponds with each reviewer’s comments.

Dear Editor,

The authors would like to thank the editor for your valuable and constructive comments. The paper has been rewritten by taking the suggestions into account.

**C1**

**When submitting your revision, we need you to address these additional requirements.**

**Please ensure that your manuscript meets PLOS ONE's style requirements, including those for file naming.**

**C2**

**We note that you have provided funding information that is not currently declared in your Funding Statement. However, funding information should not appear in the Acknowledgments section or other areas of your manuscript. We will only publish funding information present in the Funding Statement section of the online submission form.**

**Please remove any funding-related text from the manuscript and let us know how you would like to update your Funding Statement. Currently, your Funding Statement reads as follows:**

**JSPS KAKENHI Grant Number JP17J06986**

**JST, PRESTO Grant Number JPMJPR16D3**

**C3**

**We noticed you have some minor occurrence of overlapping text with the following previous publication(s), which needs to be addressed:**

**https://ieeexplore.ieee.org/document/8341739**

**In your revision ensure you cite all your sources (including your own works), and quote or rephrase any duplicated text outside the methods section. Further consideration is dependent on these concerns being addressed.**

A1-3

Thank you for your comments. We modified the format and deleted the funding information.

Dear Reviewer 1,

The authors should write the paper more explicitly. In some instances, the paper is difficult to follow. For instance, the use of different force ranges in different places.

**Q1-1**

**The abstract is difficult to read and needs to clearly portray the motivation of the study.**

A1-1

Thank you for your comment. We modified the abstract to write the motivation clearly.

**Q1-2**

**The Introduction is lacking many details to give the reader the necessary information to support the motivation for the study:**

**Q1-2-1**

**Line 18-19 – what are the physical and psychological factors impacting sense of effort?**

**Q1-2-2**

**What do the numbers reported for Stevens’ power law mean about actual perceived and executed muscular effort?**

**Q1-2-3**

**Why isn’t there a description of Weber-Fechner’s law (some readers likely do not know what this is)?**

**Q1-2-4**

**How does muscle activity change the sense of force?**

**Q1-2-5**

**Overall, the introduction just gives a list of studies, but does not provide any framework with which to understand the motivation of the authors experiments.**

A1-2-1

We believe as follows:

Physical factor : Physical features, such as the color and/or surface texture, of the objects affect the perception. For example, the smoother the surface texture, the greater is the perceived weight [1]. Because the human exerts larger force to prevent fall the object when he/she pinch the smoother surface object.

Psychological factor : Psychological factor, such as the fatigue, of the human affect the perception. For example, the perceived force/weight is become larger when human feel fatigue [2]-[4]. Because the human need to exert larger force than usual to lift the object when he/she’s muscle have fatigue.

We believe that it is caused by human’s experience or knowledge in the past.

[1] Flanagan J, Wing A, Allison S, Spenceley A. Effects of surface texture on weight perception when lifting objects with a precision grip. Perception & Psychophysics. 1995; p. 282-290.

[2] Gandevia S, McCloskey D. Sensations of heaviness. Brain. 1977;100(2):345-354.

[3] Joseph M. Muscle fatigue degrades force sense at the ankle joint. International Journal of Industrial Ergonomics. 1999;24:223-233.

[4] Nicolas V, Matthieu B. Muscular fatigue and its effects on weight perception. Gait & Posture. 2008;28:521-524.

A1-2-2

These power values represent the ratio of the intensity of the subject's perceived exertion of force to the actual exertion.

A1-2-3

Thank you for your valuable comment. We added the description of Weber-Fechner’s law.

A1-2-4

The exertional force of a muscle is determined by the motor command generated in the central nervous system, and the muscle activity reflects the magnitude of the motor command. The larger the motor command, the more power a human can exert, and the size of motor command is related to the sense of effort size [5]. Therefore, the greater the muscle activity, the greater the sense of effort.

[5] Morree H, Klein C, Marcora S. Perception of effort reflects central motor command during movement execution. Psychophysiology. 2012;49:1242-1253.

A1-2-5

Thank you for your valuable comment. We modified the introduction including the answers of A1-2-1, 2, 3, and 4.

**Q1-3**

**Line 57-59: The authors do not present sufficient evidence that “sense of force and heaviness can be estimated from muscular activity, which can be interpreted as sense of effort”- it is unclear how this statement is true given the evidence that the authors have presented.**

A1-3

We agree with your comment, the sense of effort cannot be measured directly from the magnitude of muscle activity. There is not a simple scaled relationship between muscle activation and sense of effort. However, since there is a positive correlation between the both, and it is possible to evaluate the sense of effort based on the muscle activity value, we used the muscle activity in this study. I modified the expression in that sentence.

**Q1-4**

**The authors do not present any information for the specifics of the staircase methodology.**

**Q1-4-1**

**What specific steps were used?**

**Q1-4-2**

**What happened if subjects made an error?**

**Q1-4-3**

**Why were the postures of 30 and 60 degrees chosen?**

**Q1-4-4**

**Line 11 – How were experimental stimuli modified based on the participant’s response?**

A1-4-1

There are 2 types, which are downward-step and upward-step, in which the stimulus value deviates from the standard value (very large and very small). In this case, very large/small means the subject can definitely perceive the difference from standard stimulus.

A1-4-2, A1-4-4

According to the response of each trial, the test stimulus of next trial for downward-step and upward-step was changed as follows.

- Answer that the test stimulus is greater than the reference stimulus: Reduce the test stimulus by 0.2 Nm.
- Answer that the test stimulus is smaller than the test stimulus: Increase the test stimulus by 0.2 Nm.

These procedures were used in both downward-step and upward-step and downward-step and upward-step were conducted alternately.

A1-4-3

We chose from the possible positions during driving.

All the answers of Q1-4 are reflected in the paper’s description.

**Q1-5**

**This task has a significant memory component, in that subjects have to hold the posture for 3 seconds and memorize it, how do the authors account for aspects of memory that may contribute to the task?**

A1-5

The task itself is not composed of "Memorize the complex motor task", so we believe the effect is small.

**Q1-6**

**Line 3 - Heaviness typically refers to the weight of an object (eg., holding it and testing weight in your hand). The subjects are not supporting the steering wheel, but are instead gripping the wheel in response to a force being applied. It is unclear whether the authors are testing response to heaviness or response to an applied force. As the directions they have supplied to subjects do not match up with the description of the research in the introduction.**

A1-6

The task of this study was to compare the magnitude of the force given while holding the steering wheel. The expression "Heavier" cause confusing, so we modified the expression in these sentences

**Q1-7**

**Line 114-116: The authors describe 200 trials, with 25 repetitions in each mode, but there is no description of what these modes were. Clarification and explicit statements are experimental procedures are needed here.**

A1-7

Each posture has 25 downward-step and 25 upward-step. So, 4 postures × (25 + 25 trials) = 200 trials.

We added this explanation in the paper.

**Q1-8**

**Why wasn’t EMG collected and then confirmed via the model-based approach?**

A1-8

One of the drawbacks of surface electromyogram measurement (sEMG) is that its surface electrodes have a limited spatial resolution and detect, therefore, only a superposition of a very large number of muscle motor units. The separation of the activity of single motor units from simultaneous adjacent ones is hard to achieve with surface electrodes and needs accurate measurement and complicated signal processing [6]. Moreover, sEMG recordings can indicate when a muscle is active, but examination of sEMG recordings does not allow one to determine which motions of the body arise from a muscle’s activity [7].

Since it is possible to estimate the activity of multiple muscles, especially muscles in deep, by using the simulation using the musculoskeletal model, we used the simulation in this experiment. Of course, there are some problems in the simulation such as the consistency of the results and the accuracy of model. In the future, we intend to carry out experimental design while taking both advantages.

[6] Rau G., Schulte E., Disselhorst-Klug, C., From cell to movement: to what answers does EMG really contribute?, Journal of Electromyography and Kinesiology, 2004;14:611-617.

[7] Delp, S. L., Anderson, F. C., Arnold, A. S., Loan, P., Habib, A., John, C. T., Guendelman, E., and Thelen, D. G., Opensim: Opensource software to create and analyze dynamic simulations of movement, IEEE Transactions on Biomedical Engineering, 2007;54(11):1940-1950.

**Q1-9**

**The utilization of parameters proposed by Thelen is surprising given that this original citation examined age-related changes in biomechanics from age 30-70 (young vs old adults). The sample here is roughly ten years younger and it is likely that these same parameters do not apply to younger adults.**

A1-9

Yes, Thelen's muscle model is for older adult, as you pointed out. We made mistake in the reference. The musculoskeletal model and muscle parameter are used from Holzbaur et al. for the musculoskeletal simulation in this study, as described in the section "Muscle activity estimation using 3D musculoskeletal model". Each parameter of this musculoskeletal model uses the values described in the paper [8] - [12]. Since these papers do not specify age (survey of cadaver specimens), the average of the parameters from 5 papers is used for the musculoskeletal model.

We modified the expression in these sentences

[8] 2An, K. N., F. C. Hui, B. F. Morrey, R. L. Linscheid, and E. Y. Chao.Muscles across the elbowjoint:Abiomechanical analysis. J. Biomech. 1981;14:659–669.

[9] 22Jacobson, M. D., R. Raab, B. M. Fazeli, R. A. Abrams, M. J. Botte, and R. L. Lieber. Architectural design of the human intrinsic hand muscles. J. Hand Surg. [Am.] 1992;17:804–809.

[10] Langenderfer, J., S. A. Jerabek, V. B. Thangamani, J. E. Kuhn, and R. E. Hughes. Musculoskeletal parameters of muscles crossing the shoulder and elbow and the effect of sarcomere length sample size on estimation of optimal muscle length. Clin. Biomech. 2004;19:664–670.

[11] Lieber, R. L., B. M. Fazeli, and M. J. Botte. Architecture of selected wrist flexor and extensor muscles. J. Hand Surg. [Am.] 1990;15:244–250.

[12] Lieber, R. L., M. D. Jacobson, B. M. Fazeli, R. A. Abrams, and M. J. Botte. Architecture of selected muscles of the arm and forearm: Anatomy and implications for tendon transfer. J. Hand Surg. [Am.] 1992;17:787–798.

**Q1-10**

**The results section of this manuscript is two small paragraphs. This gives the reader very little information to go on in terms of what the outcome of this study is and how it relates to their hypotheses and framework.**

A1-10

Thank you for your valuable comment. We modified the results session.

**Q1-11**

**Perceptual bias needs to be clearly defined in the abstract**

A1-11

Thank you for your comments. We added the explanation of perceptual bias in the abstract.

**Q1-12**

**9 subjects seems like far too few for a study like this. Is this study appropriately powered? Why was one of the subjects unable to drive? Does this subject have extensive driving experience?**

A1-12

We believe the number of subjects is enough because we got the statistical difference significantly. And since the results shows the bias, we believe that the experiment was conducted properly.

One of the subjects cannot drive in real life because he does not have a driving license. We allow all participants practice the task before the experiment. And since the experimental task do not require the driving skills and subjects are healthy young adult.

**Q1-13**

**Line 113 – How long were the breaks that subjects were allowed?**

A1-13

If the subjects reported fatigue, we allowed them to rest until the fatigue disappeared.

**Q1-14**

**Why weren’t the experimental stimuli evenly counterbalance. It is possible that presenting with more “heavier” trials will bias subjects to over-estimate, based on previous history of stimuli.**

A1-14

Thank you for your valuable comment. There are two modes: the downward step, in which the experimental stimulus value starts from a value larger than the reference stimulus value, and the upward step, in which the experimental stimulus value starts from a smaller value.

In the experiment, the downward and upward step sequences alternate. As described above, the stimulus changes according to the response. (For example, Reference stimulus -> Downward stimulus -> Answer -> Reference stimulus -> Upward stimulus -> Answer -> Reference stimulus -> Downward stimulus (considering the answer from last downward stimulus) -> Answer -> Reference stimulus -> Upward stimulus (considering the answer from last upward stimulus) -> Answer ->…). Then the experimental stimulus is repeated at the comparative stimulus of the chance level, that is, near the subjective equivalence value. Therefore, the bias can be determined with high accuracy.

We modified the description of the experiment in the paper.

**Q1-15**

**Figure legends need more description.**

A1-15

Thank you for your valuable comment. We modified the figure legend.

Dear Reviewer 2,

The authors should write the paper more explicitly. In some instances, the paper is difficult to follow. For instance, the use of different force ranges in different places.

**Q2-1**

**First of all, the authors report that they investigate effects of force. However, since the task concerns a rotation of the steering wheel, this is actually about torques, not forces. Indeed, the biases are reported in Nm, not N. Although the participants are asked to report which stimulus was heavier, the authors should clarify that they measure torques.**

A2-1

We assume that the subject is not exerting a torque to rotate the steering because the subject perceives the force while maintaining the steering angle. We believe that subjects perceive the tangential direction force of the circle by maintaining the posture as shown in the figure below.

**Applied force**

**Exerted force**

**: Hand position**

**Steering wheel**

**Q2-2**

**One main concern about the methods is that the different postures also involve different torque directions. I see that this was done to have realistic conditions in driving and steering, but this could influence the results. In fact, it seems that the different orientations do not differ in perceptual bias (i.e. no difference between 30 and 60 degrees, neither between -30 and -60 degrees), but the bias direction (i.e. negative or positive) is different. So the biases also seem to be driven by the direction of the torques. By altering the direction, the authors seem not to have not only investigated the effect of posture (with different muscle contributions) on force perception, but also the effect of torque direction (which should also lead to different muscle contributions based on the direction of the force that is applied by the arm) on perception. Since the same torque direction was used in the reference as the experimental condition, this still investigates an effect of posture for each condition on its own. Therefore, I think the results are still interesting, since significant biases were found, but only postures with the same force direction can be compared. In sum, I think the authors should split their analysis by the two torque directions.**

A2-2

Yes, the direction of the force also has an influence on the force perception. As described in the discussion, it has been reported that the difference between the direction of gravity and anti-gravity force affects the force perception [10]-[14]. However, previous studies have not investigated the physical characteristics of people, and this study focuses on muscle activity. The results showed that muscle activity had some influence on perception. Changes in posture can also be considered as one of the factors that changes muscle activity. In future work, we would like to increase the number of experimental postures and examine the effects of postures.

[10] Sakajiri T, Tanaka Y, Sano A. Relation between gravitational and arm-movement direction in the mechanism of perception in bimanual steering. Experimental Brain Research. 2013;231:129-138. doi:10.1007/s00221-013-3676-0.

[11] Hood B. Gravity rules for 2- to 4-year olds? Cognitive Development. 1995;10:577-598. doi:10.1016/0885-2014(95)90027-6.

[12] Winter J, Allen T, Proske U. Muscle spindle signals combine with the sense of effort to indicate limb position. The Journal of Physiology. 2005;568(Pt 3):1035-1046. doi:10.1113/jphysiol.2005.092619.

[13] Ross H, Brodie E, Benson A. Mass-discrimination in weightlessness and readaptation to earth's gravity. Experimental Brain Research. 1986;65:358-366. doi:10.1111/1467-7687.00051.

[14] Young L, Oman C, Merfeld C, Watt D, Roy S, DeLuca C, et al. Spatial orientation and posture during and following weightlessness: human experiments in Spacelab Life Sciences 1. Journal of Vestibular Research. 1993;3:231-239.

**Q2-3**

**Related to this, I think the authors should report the muscle activity for the reference condition as well. These were performed with two different torque directions. If the muscle activity differs between these directions in the same posture, this would already show that the muscle activity also varies because of torque direction effects, not only posture.**

A2-3

Thank you for your valuable comment. We agree that the comparison between reference force can discuss deeply about force direction. We added the figure in the paper. The Welch’s t-test showed a significant difference between the directions of the force, indicating that the direction of the force has a significant effect.

*** : p < 0.001

10

***

0

5

Muscle activity [%]

Gravitational

Anti-gravitational

(-30, -60 degree)

(30, 60 degree)

Force direction

**Q2-4**

**Another concern I have about the methods is the muscle model. To me, it seems strange that the muscle activity of agonist and antagonists are averaged. They mention this as a limitation in the discussion, but I think the muscle model as it is now is not very insightful. The average activity of multiple muscles can give similar values for very different combinations of different muscles. Perhaps they could calculate the activity for different muscles (or at least only average over agonist and antagonist muscles). If the activity pattern is similar in different tasks and only increases overall, this would validate their decision to average the muscle activity, but now this is unknown.**

A2-4

In this study, the muscle activity is considered as the total amount of sense of effort, and therefore we used the average activity of the representative muscles during steering. I agree your comment, muscles have agonist and antagonist muscles for certain movement. In the future, we would like to evaluate the sense of effort considering the muscle activity pattern.

**Q2-5**

**It seems to me that the calculations for the predicted bias are unnecessarily complicated. I do not think the perception-change ratio is needed to do this. When equation 5 and 7 are combined: Fp=a log (alpha-m/k)+b. Therefore, equation 6, 8 and 9 do not seem necessary.**

A2-5

Thank you for your valuable comment. We modified these equations.

**Q2-6**

**I do not understand where the values in Table 1 come from. Are they from a previous paper or are they fitted to the data? If the latter is true, what data is exactly compared to which model to obtain these parameter values?**

A2-6

Yes, we used the parameter from previous paper in [13]. We used the parameters of a past experiment in which the steering force perception.

[13] Kishishita Y, Takemura K, Yamada N, Hara T, Kishi A, Nishikawa K, et al. Prediction of Perceived Steering Wheel Operation Force by Muscle Activity. IEEE Transactions on Haptics. 2018;11:590-598.

**Q2-7**

**The authors correlate the predicted biases from the muscle activity/measured forces to the measured biases. However, I wonder whether they can do it the way they have done it now.**

**First, similarly to my earlier comment about comparisons between the torque directions, it might not be fair to correlate over two different torque directions. The biases in one direction are negative, while the biases in the other direction are positive. I am not sure whether it is justified to combine these two conditions into a single plot.**

**Secondly and related to this, the authors seem to have combined different data points (for different conditions) for the same subjects into one plot. Different conditions for one subject are within factors, whereas data points for different subjects have between factors and therefore have different sources of variation. I am not an expert on these statistics, but I wonder whether this can be combined in a single correlation. Perhaps a multilinear regression model might be more suitable.**

A2-7

Yes, the result of the bias prediction from different force directions are included in the same graph (Fig. 6). However, in this prediction method, the bias is predicted using the muscle activity regarded as the sense of effort size. We believe that the effects of different force directions are reflected by differences in the magnitude of muscle activity. Therefore, we believe that plotting on the same graph is not a problem.

**Q2-8**

**Do the authors have an explanation why they do not find a bias for the 60 degree condition? This does not follow from the muscle activity pattern, so perhaps the authors could indicate other factors that drive the perception?**

A2-8

The results in Fig. 4 show that the bias is almost zero, but as shown in the following figure, the bias is divided into positive and negative directions, and then the average becomes zero because of the large standard deviation. Although the muscle activity did not increase largely from 30° to 60°, the negative bias is occurred in 60° due to the different posture. It is possible that the change in posture affected the skill of the subject's movements, which changed the bias direction. However, since another experiment is needed to investigate the skill of movement, we will conduct in future work.

AVG.

**Q2-9**

**P2 L46 I am not sure whether visual information can be considered a cognitive feature. This could also be more low-level sensory effects that contribute to the ultimate heaviness perception.**

A2-9

Thank you for your valuable comment. It is inappropriate to claim that visual information is a cognitive feature. In that paper, they report that perceptual bias is generated when a visual delay is added to an object lifting motion in virtual reality environment. And they have mainly discussed about multisensory information affect the weight perception.

We modified the sentence about that in the paper.

**Q2-10**

**The authors report that they counterbalance the conditions to avoid order effects. However, it seems that the reference condition was always performed before the experimental condition. Could this not also lead to order effects or biases? See for example the time-order error as described by Hellstrom (1985, Psychological Bulletin; 2003 Perception & Psychophysics)**

A2-10

Thank you for your valuable comment. We didn’t consider time-order error in this experiment. However, if there is a time-order bias, the reference stimulus (reference stimulus was presented at first in all experiments) is underestimated in any posture. According to the experimental results, the bias direction is different depending on the experimental conditions, and we believe the effect of time-order error is small. And, in the experiment, the downward and upward step sequences alternate. As described above, the stimulus changes according to the response.

(For example, Reference stimulus -> Downward stimulus -> Answer -> Reference stimulus -> Upward stimulus -> Answer -> Reference stimulus -> Downward stimulus (considering the answer from last downward stimulus) -> Answer -> Reference stimulus -> Upward stimulus (considering the answer from last upward stimulus) -> Answer ->…).

Then the experimental stimulus is repeated at the comparative stimulus of the chance level, that is, near the subjective equivalence value. Therefore, the bias can be determined with high accuracy.

We modified the description of the experiment.

**Q2-11**

**P5 L158 The authors state that they test whether the bias was different between the standard and experimental procedure. However, from the psychometrical curve, the bias is defined as the difference between the standard and the experimental stimulus, so there can be no bias calculated for the standard position. Do the authors mean they tested whether the biases were significantly different from zero?**

A2-11

Yes, the bias values for nine subjects in each position were compared with 0° to perform the t-test.

**Q2-12**

**P6 L195 I think this should be Eq. 8, not Eq. 1?**

**Q2-13**

**The author contributions are not described (P 9).**

**C2-1,2,3**

**- P1 L18 "..to sense of force..." remove "of"**

**- P1 L24 "Thus" seems strange here, since fatigue affects do not relate to the previous sentence about colour affects. Perhaps replace with "In addition".**

**- P5 L178 "ANOVA was performed" -> An ANOVA was performed**

A2-12, 13, C2-1, 2, 3

Thank you for your valuable comment. We modified these sentences.
